# Mycotic Aneurysms and Recurrent Intracranial Hemorrhages in a Patient With Infective Endocarditis

**DOI:** 10.7759/cureus.32591

**Published:** 2022-12-16

**Authors:** Usama Talib, Muhammad Akash, Sushritha Reddy, Sania Ayub, Hadeel Dawoud, Ahmed H Abdelfattah

**Affiliations:** 1 Internal Medicine, University of Kentucky College of Medicine, Lexington, USA; 2 Internal Medicine Resident, MGB-Salem Hospital, Salem, USA; 3 Internal Medicine, Malla Reddy Institute of Medical Sciences, Hyderabad, IND; 4 Internal Medicine, Quetta Institute of Medical Sciences, Quetta, PAK; 5 Critical Care Medicine, Mansoura University, Mansoura, EGY; 6 Hospital Medicine, University of Kentucky College of Medicine, Lexington, USA

**Keywords:** goals of care, septic emboli, transthoracic echocardiography (tte), subarachnoid hemmorhage, myo-pericarditis, infective endocarditis, intracranial hemorrhage (ich), mycotic aneurysms

## Abstract

The increase in the use of IV drugs has been accompanied by an increase in the incidence of infective endocarditis (IE). The clinical picture, vitals, examination, blood cultures, laboratory tests, and imaging can help diagnose IE. The Duke criteria also play a role in the diagnosis of IE. Prolonged antibiotic use and even interventions may be needed in the management of specific cases. Rare complications such as mycotic aneurysms and intracranial hemorrhages can be fatal and must be promptly addressed to prevent loss of life and serve debilitation in these patients.

## Introduction

The term infective endocarditis (IE) refers to the infection of the endothelium of the heart, that is, the heart valves. The incidence of IE is estimated to be between 3-10 per 100,000 individuals in the general population and 150-200 per 100,000 individuals among those with intravenous drug use (IVDU) [1]. Common risk factors for IE include IVDU, indwelling intravenous (IV) catheters, intracardiac devices, immunocompromised status, and recent dental/surgical procedures [2]. The incidence of IE associated with IVDU has been on the rise in North America [3]. The common presentation includes a history of IVDU or dental infection/procedure with recent fevers, fatigue, and weakness. The diagnosis of IE can be made by using the Duke criteria. The diagnosis of IE requires obtaining proper patient history including the history of IVDU, clues on physical examination (such as IV injection marks, new cardiac murmur, petechiae, splenomegaly), and laboratory as well as imaging evidence [blood cultures, CT scan, and transthoracic echocardiogram (TTE)]. A new atrioventricular (AV), fascicular block on the EKG of a patient with IE is indicative of perivalvular extension of infection, possibly a perivalvular abscess [2]. Patients with suspected IE on admission should have blood cultures obtained to identify the causative organism. IE can be classified into culture-positive (blood cultures are positive in 98% of IE cases) and culture-negative IE (high index of suspicion with negative blood cultures) [4]. Central nervous system (CNS) involvement has also been observed in IE.

Antibiotics (primarily IV) are the mainstay of treatment of IE, but treatment may vary based on the presence of complications. Cardiac complications are more common (approaching 50%), whereas symptomatic CNS complications have been reported to be around 15-30% [4]. Mycotic aneurysms from IE are estimated to be between 0.7 and 4% and carry a high risk of mortality if not promptly addressed. The ruptured mycotic aneurysm is associated with a reported mortality rate of around 80% [4]. An understanding of mycotic aneurysms and intracranial bleeding with the associated high risk of morbidity and motility is required to raise awareness to educate patients more effectively as well as to perform diagnostic tests in a prompt manner to diagnose such complications and address them. A multidisciplinary approach is required to handle complications of endocarditis including persistent bacteremia despite IV antibiotic use, heart blocks, mycotic aneurysms, cavitary lung lesions, and intracranial bleeding [4].

We present a case of IE in a young patient with a history of IVDU that manifested as weakness and altered mentation with the hospital course complicated by the identification of mycotic aneurysms, intracranial bleeding, and associated seizures, ultimately resulting in the demise of the patient.

## Case presentation

**Figure 1:**
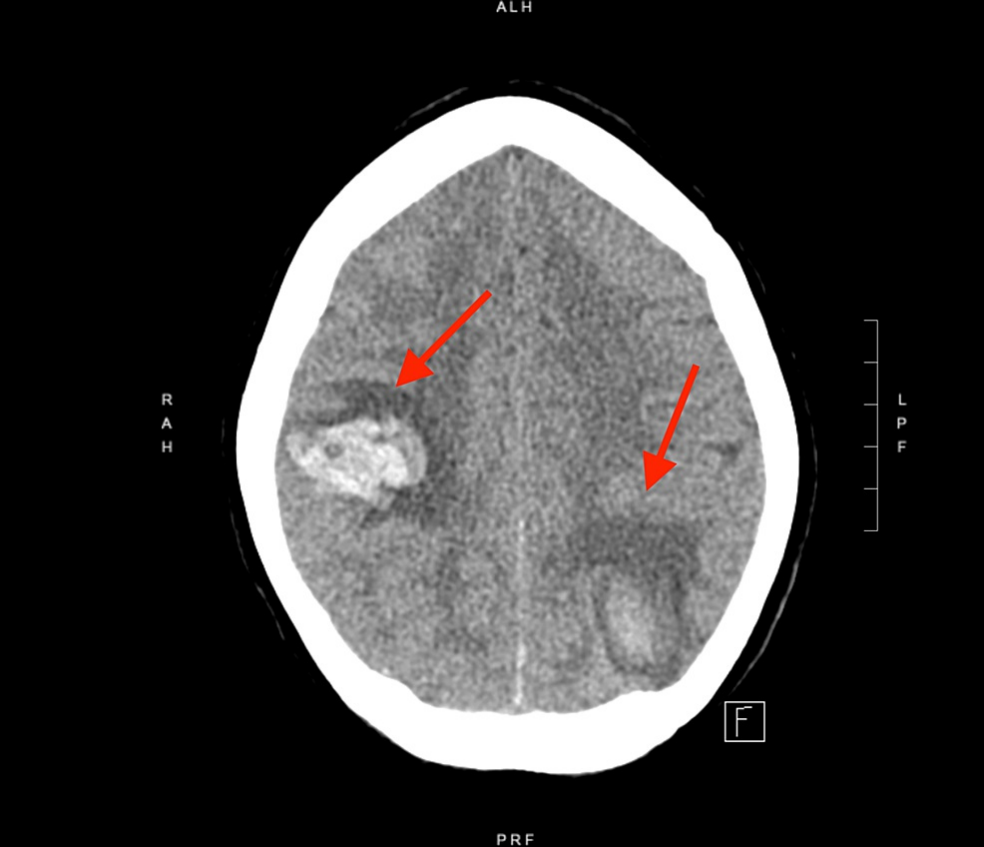
CT scan of the head Red arrows depict the right and left parietal foci of hemorrhages CT: computed tomography

A 32-year-old female presented with sudden-onset left upper extremity weakness, altered mentation, and generalized headache. Her past medical history was significant for mitochondrial myopathy resulting in wheelchair dependence, nicotinamide adenine dinucleotide dehydrogenase (NADH) deficiency, and IVDU, with the patient unable to specify the timing of the most recent use. On admission, she was afebrile, tachycardic, and tachypneic. Physical examination was significant only for left upper extremity weakness and injection marks in the right antecubital fossa. CT head without contrast showed peripherally located parenchymal hemorrhages in the right frontoparietal region and left parietal lobe and adjacent parenchymal hyperdensities concerning for edema or ischemia as well as punctate nonspecific calcific density along the periphery of right frontal parietal hemorrhage (Figure 1).

**Figure 2:**
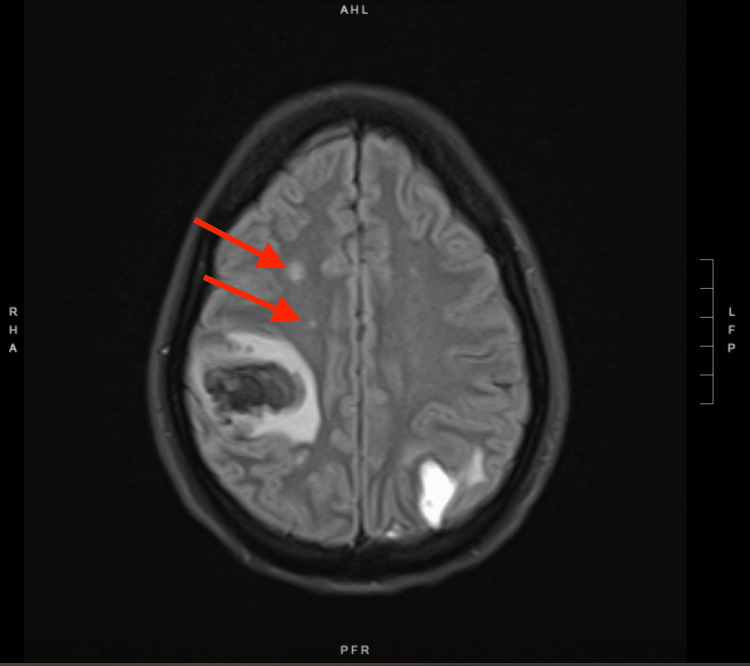
MRI brain Red arrows depict foci of punctate enhancement. Areas of corresponding FLAIR signal abnormality in the bilateral cerebral hemispheres are also visible MRI: magnetic resonance imaging; FLAIR: fluid-attenuated inversion recovery

**Figure 3:**
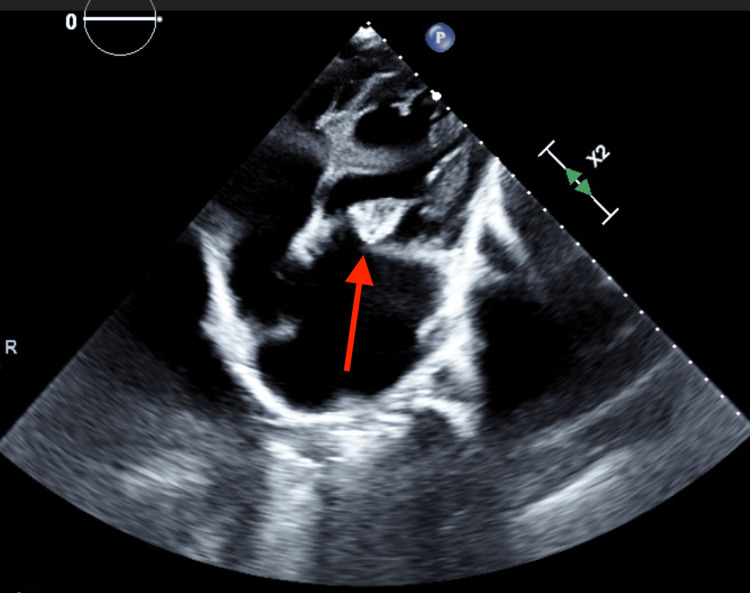
TTE The red arrow indicates mitral valve vegetation TTE: transthoracic echocardiogram

MRI brain showed additional multiple enhancing lesions throughout supratentorial regions bilaterally, which were concerning for metastatic disease (Figure 2). CT scans of the chest, abdomen, and pelvis were done and showed heterogeneous enhancing lesions in the pancreatic tail and splenic hilum, and a 7-mm noncalcified nodule in the right middle lobe of the right lung with borderline mediastinal lymph nodes. This raised a concern for metastasis versus disseminated infection in the setting of the patient's IVDU. The patient did not have a personal or family history of cancer. Also, there was no history of unintentional weight loss or B-symptoms, making the diagnosis of cancer less likely. Blood cultures that were sent on admission returned positive for Streptococcus salivarius and Streptococcus sanguinis. A TTE revealed thickened mitral valve leaflets with a mobile, echogenic, irregular mitral valve mass on the anterior leaflet consistent with vegetation (Figure 3). The patient fulfilled the requirement for a diagnosis of IE based on the Duke criteria as she had two positive major criteria (1: vegetation on TTE and 2: positive blood cultures for streptococcus) and three minor criteria (1: injection drug use, 2: fever, and 3: vascular phenomena including intracranial hemorrhage and mycotic aneurysms) and was hence conclusively diagnosed to have IE.

**Figure 4:**
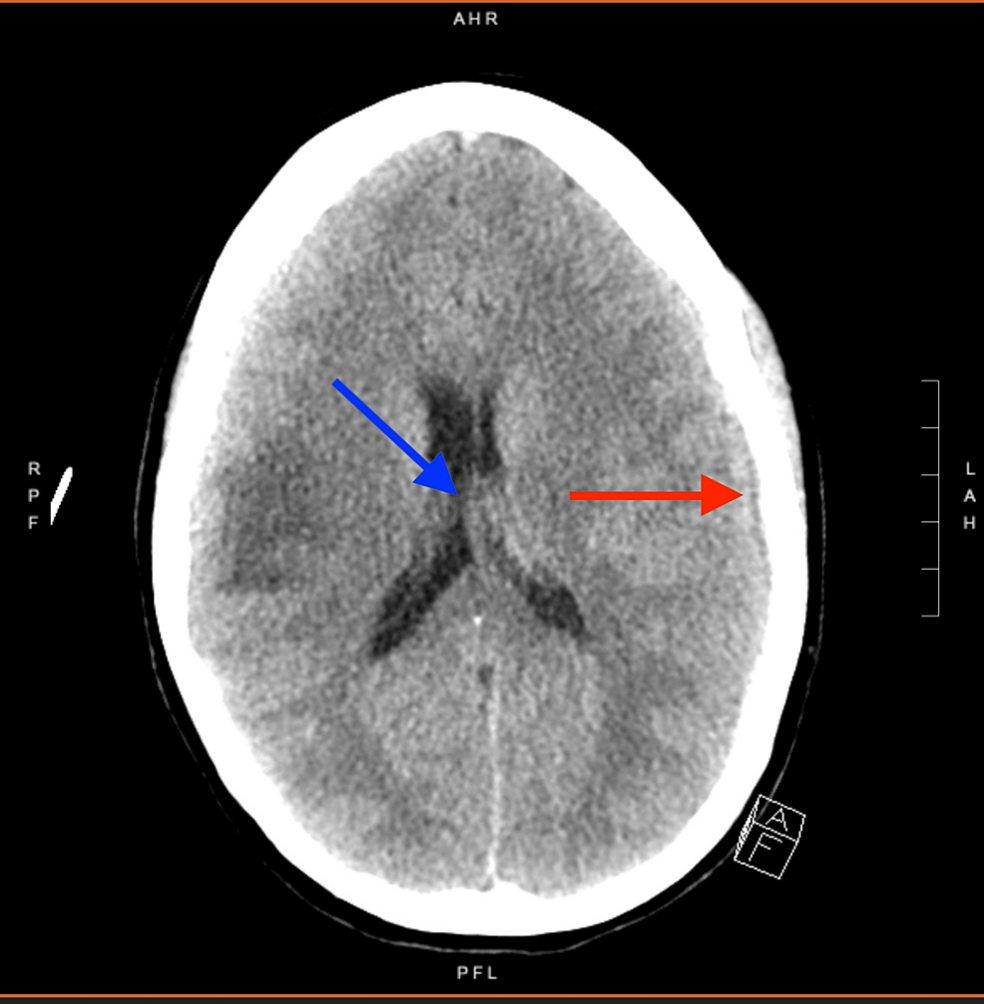
Subdural hematoma (red arrow) and midline shift (blue arrow)

The infectious disease (ID) team was consulted, and the patient was started on IV antibiotics with a plan to continue antibiotics for four to six weeks, with a possible transition to oral antibiotics after the initial IV treatment. Neurosurgery, neurology, and cardiology teams assisted with the management of the patient. The neurosurgery team, on initial evaluation, did not recommend neurosurgical intervention as the lesions appeared most likely to be subacute-chronic hematomas. On day four of hospitalization, the patient developed acute-onset substernal chest pain with a significant rise in troponin levels, but no changes were noted on EKG. A bedside echo was negative for any new wall motion abnormalities or acute pathology. CT chest did not show coronary artery calcifications or pulmonary embolism (PE). Myopericarditis was suspected, and she was started on colchicine and ibuprofen with the approval of the neurosurgical team. On day eight of hospitalization, the patient developed sudden right facial droop, dysarthria, and change in mentation. CT head was repeated and showed new left cerebral convexity subdural and subarachnoid hemorrhages with increased mass effect with 4-mm rightward midline shift and increased diffuse sulcal and basal cistern effacement (Figure 4). It also revealed evolving intraparenchymal hemorrhages in the bilateral cerebral hemispheres with unchanged surrounding vasogenic edema. Cardiothoracic surgery was consulted but the patient was not considered a surgical candidate due to neurological deterioration given recurrent intracranial hemorrhages and worsening mycotic aneurysms needing neurosurgical monitoring.

The neurosurgery team recommended head and neck CT angiography (CTA), which showed no significant stenosis within the cervical carotid and vertebral systems. It also revealed two 1-2-mm hyperattenuating foci along the posterior margin of the right parietal intraparenchymal hemorrhage, one likely representing a calcification, and the other suspicious for mycotic aneurysm arising from distal right middle cerebral artery (MCA) branches. Goals of care discussions resulted in the patient opting for a "do not resuscitate/do not intubate" (DNR/DNI) status. 

Angiography and embolization of the mycotic aneurysm were performed by the neurosurgery team. There was a concern for some contrast extravasation into the Sylvian fissure but repeat CT head did not show any new findings. The patient continued to have mild weakness in her right upper limb, as well as aphasia, right facial droop, and episodes of brief self-terminating tonic clinic seizures. EEG showed focal sharp waves and the patient was started on antiepileptics per neurology recommendations. She continued to have worsening mental status, with a new infarction in the left frontal lobe in the region of a known mycotic aneurysm. The multidisciplinary meeting concluded that no further intervention would provide a meaningful outcome for the patient. Hospice was consulted due to her deteriorating health and poor prognosis. Goals of care discussions led to the decision to not pursue aggressive therapy and instead pursue comfort measures in accordance with the wishes of the patient. The patient passed away in the presence of her family and loved ones on day 16 of hospitalization.

## Discussion

IE is more common among those with active IVDU, and the incidence of IE has been on the rise [1,3]. Other than IVDU, indwelling IV catheters, intracardiac devices, immunocompromised status, and recent dental/surgical procedures are known risk factors for IE [2].

Diagnosis of IE requires obtaining appropriate patient history, physical examination, laboratory tests as well as imaging evidence. Blood cultures are collected before the initiation of antibiotics. TTE is the initial modality of choice to assess for the presence of vegetation in suspected cases. In the case of bradycardia in IE patients, EKG can reveal AV nodal and fascicular blocks suggestive of perivalvular extension of infection with concern for perivalvular abscess [2]. Blood cultures are usually positive, but culture-negative IE is being increasingly recognized. This may be seen in patients with symptoms suggestive of IE (and diagnosis with Duke/modified Duke criteria) but a negative TTE [5]. A transesophageal echocardiogram (TEE) or a CT/PET can be obtained in cases of negative TTE with high suspicion of IE and to assess for possible complications [4].

Patients with IE can present with symptoms such as fever, night sweats, and malaise [2]. Our patient had an atypical initial presentation with stroke-like symptoms, but given her history of IVDU, she was a high risk for IE. This highlights that patients can present with any of the complications of IE, and it should warrant simultaneous management of the complication along with investigation for underlying IE. Potential complications of IE include heart failure, atrioventricular block, perivalvular abscess, pericarditis, and septic embolization to systemic and pulmonary circulation. CNS involvement has been reported in 30-65% of the cases [1,6]. Symptomatic CNS complications have been reported in 15-30% of cases [4]. Embolization to the vessels in arterial walls, i.e., vasa vasorum, most commonly branching points, can lead to the formation of mycotic aneurysms [2].

Mycotic aneurysms are not commonly seen in IE, but the overall prevalence is not known. The prevalence of cerebral mycotic aneurysms is 0.7-4% among all patients with cerebral aneurysms [4,7]. Mortality rates have been found to be almost 30% for unruptured mycotic aneurysms and as high as 80% for ruptured mycotic aneurysms [8].

Management of cerebral mycotic aneurysms should involve discussion involving a multidisciplinary team including internal medicine, infectious disease, cardiology, neurology, neurosurgery, and interventional radiology. Different treatment options include IV antibiotics with or without an endovascular/surgical approach [4]. Cardiac lesions can be addressed by surgical or non-surgical methods. The AngioVac System (AngioDynamics, Latham, NY), comprising a percutaneous aspiration device, has been increasingly getting attention for right-sided IE [9]. In case of ruptured mycotic aneurysms, urgent invasive management is indicated. In the absence of evidence of intracranial hemorrhage from mycotic aneurysms, surgical risk should be assessed [10]. In cases of high surgical risk, a conservative approach is preferred but an invasive approach can be taken on a case-by-case basis. In cases of low to medium surgical risk, an invasive approach should be preferred [10].

Our patient developed an intracranial bleed requiring angiography and embolization of a ruptured mycotic aneurysm. However, she continued to have recurrent intracranial bleeding related to mycotic aneurysms, ultimately leading to her demise.

## Conclusions

We discussed the case of a patient with multiple ruptured mycotic aneurysms secondary to IE from IVDU, which has rarely been reported in IE patients. We emphasize the importance of timely diagnosis and management of IE (medical and interventional/surgical) to minimize its catastrophic complications.
